# The impact of myeloproliferative neoplasms (MPNs) on patient quality of life and productivity: results from the international MPN Landmark survey

**DOI:** 10.1007/s00277-017-3082-y

**Published:** 2017-08-05

**Authors:** Claire N. Harrison, Steffen Koschmieder, Lynda Foltz, Paola Guglielmelli, Tina Flindt, Michael Koehler, Jonathan Mathias, Norio Komatsu, Robert N. Boothroyd, Amber Spierer, Julian Perez Ronco, Gavin Taylor-Stokes, John Waller, Ruben A. Mesa

**Affiliations:** 1 Guy’s and St Thomas’ NHS Foundation Trust, Guy’s and St Thomas’ Hospital, London, SE1 9RT UK; 2 Department of Hematology, Oncology, Hemostaseology, and Stem Cell Transplantation, Faculty of Medicine, RWTH Aachen University, Aachen, Germany; 3 St Paul’s Hospital, University of British Columbia, Vancouver, BC Canada; 4 CRIMM, Center for Research and Innovation of Myeloproliferative Neoplasms, AOU Careggi, Department of Experimental and Clinical Medicine, University of Florence, Florence, Italy; 5 Patient advocate, Prato, Italy; 6 Department of Hematology and Oncology, Faculty of Medicine, Otto-von-Guericke University Magdeburg, Magdeburg, Germany; 7 MPN Voice, London, UK; 8 Department of Hematology, Juntendo University Faculty of Medicine, Tokyo, Japan; 9 Novartis Pharmaceuticals Corporation, East Hanover, NJ USA; 10 Novartis Pharma AG, Basel, Switzerland; 11 Adelphi Real World, Bollington, UK; 12 Mayo Clinic, Scottsdale, AZ USA

**Keywords:** (4–6): MPN, Quality of life, Symptom burden, Work productivity, Activities of daily living

## Abstract

Myelofibrosis (MF), polycythemia vera (PV), and essential thrombocythemia (ET) are myeloproliferative neoplasms (MPNs) associated with high disease burden, reduced quality of life (QOL), and shortened survival. To assess how MPNs affect patients, we conducted a global MPN Landmark survey. This online survey of patients with MPNs and physicians was conducted in Australia, Canada, Germany, Japan, Italy, and the United Kingdom. The survey measured MPN-related symptoms and the impact of MPNs on QOL and the ability to work as well as disease-management strategies. Overall, 219 physicians and 699 patients (MF, *n* = 174; PV, *n* = 223; ET, *n* = 302) completed the survey; 90% of patients experienced MPN-related symptoms. The most frequent and severe symptom was fatigue. Most patients experienced a reduction in QOL, including those with low symptom burden or low-risk scores. A substantial proportion of patients reported impairment at work and in overall activity. Interestingly, physician feedback and blood counts were the most important indicators of treatment success among patients, with improvements in symptoms and QOL being less important. Regarding disease management, our study revealed a lack of alignment between physician and patient perceptions relating to communication and disease management, with patients often having different treatment goals than physicians. Overall, our study suggested that therapies that reduce symptom burden and improve QOL in patients with MPNs are crucial in minimizing disease impact on patient daily lives. Additionally, our findings showed a need for improved patient-physician communication, standardized monitoring of symptoms, and agreement on treatment goals.

## Introduction

Myelofibrosis (MF), polycythemia vera (PV), and essential thrombocythemia (ET) are myeloproliferative neoplasms (MPNs) [1, 2], with global incidence rates of 0.3–1.5 [3–5], 1.5–2.0 [4–6], and 1.03–2.5 per 100,000/year, respectively [3–5]. These hematopoietic stem cell disorders are characterized by clonal proliferation of ≥ 1 cell type of the myeloid lineages [1, 2, 7] and are associated mostly with mutations in the Janus kinase 2 (*JAK2*) [8–10], calreticulin (*CALR*) [11, 12], or thrombopoietin receptor (*MPL*) genes [13, 14]. Clinical manifestations can vary by MPN subtype and can include polycythemia, anemia, leukocytosis, thrombocytosis, fatigue, and hepatosplenomegaly [9, 15, 16]. In general, patients have an increased risk of thrombotic and thromboembolic events [17] and have a higher risk of mortality compared with the general population [18–22]. Progression to MF (for those with PV or ET) or acute myeloid leukemia remains a great concern among patients [8, 23].

MPNs are associated with a substantial disease burden, often leading to a reduced quality of life (QOL) for many patients [15, 24–27]. Symptoms may include fatigue, pruritus, night sweats, microvascular symptoms, splenomegaly, and splenomegaly associated symptoms (e.g., abdominal pain, early satiety), with fatigue being one of the most severe symptoms [15, 25–27]. Among patients with MF, PV, or ET, patients with MF generally have the highest symptom burden and the lowest QOL [15].

Until recently, few reports had been published regarding patient perception of how MPNs and associated symptom burden affected their daily life and productivity at work [24]. The US MPN Landmark Survey was the first large observational study to evaluate the patient-reported impact of MPNs on overall health and productivity in contemporary patient populations in the USA [23]. This study found that symptom burden among patients with MPNs is substantial and negatively affects QOL, daily living, and the ability to work and/or be productive. Notably, this negative effect was also observed among patients with low prognostic risk scores and low symptom burden. Overall findings from this study suggested that, among other goals of therapy, treatment for MPNs should reduce symptom burden and improve QOL and productivity to enhance the overall health of patients with MPNs.

To investigate how patients with MPNs who live outside the USA are affected by the disease, we conducted a global MPN Landmark survey. Here we present the first analysis of this global survey.

## Methods

### Survey instrument

The Landmark health survey was a multi-country, cross-sectional survey of patients diagnosed with MPN and treating physicians conducted from April 2016 to October 2016. The two components (physician survey and patient survey) were conducted as separate surveys, and there was no linkage between patient and physician responses. The physician and patient surveys included 49 and 63 questions (some with multiple parts), respectively, were administered online, and required approximately 25–30 min to complete. The patient survey covered six domains: physician-patient relationship, attitudes toward disease and treatment, treatment and drug utilization, burden of disease, disease characteristics, and demographics, with various topics in each domain. Results presented relate to patient experience and resolution of symptoms, the emotional and physical impact of MPNs, and the work and activity impairment associated with MPNs. Findings from the physician survey are also reported.

### Study population

Patients diagnosed with MF, PV, or ET who were ≥ 18 years of age were eligible to take the survey. Patients participating in randomized controlled trials were excluded. Patients were recruited using two approaches. In Australia, Germany, Italy, Japan, and the UK, physicians recruited patients during normal consultations and provided patients with a recruitment letter that contained either a link to the survey, or in some cases, a phone number to contact a local fieldwork partner who then provided the survey link. Patients in Canada, Italy, Germany, and the UK were also recruited by patient organizations, which disseminated the survey links to patients. Both routes of recruitment ensured respondent anonymity, and the route of recruitment was identified via the patient unique link number. Fully de-identified respondent information was collated and aggregated by local fieldwork partners and anonymized survey links. Physicians who were actively managing patients with MPNs were recruited via fieldwork agencies based in each country. The agencies provided them with a link to access the physician survey.

### Statistical analyses

Analyses used descriptive statistics, and no formal hypothesis was tested. The reported statistics depended on the type of variable described. For numerical variables, the respondent base, mean, and range (minimum and maximum values) were reported. For categorical variables, the respondent total and number and percentage of responses are shown. Subgroup analyses, including age, sex, prognostic risk score, and overall symptom burden, were also performed. Symptom severity was assessed by quartiles (Q1-Q4). When mean scores are reported, student *t* tests were performed on them; only statistically significant findings are referenced.

### Study oversight

A steering committee was recruited consisting of local medical experts from each participating country, except Australia, and patient organization leaders from select countries. The steering committee contributed to the survey methodology and material design before submission to the central ethics review board, Freiburger Ethik-Komission International. The physician survey received approval on April 4, 2016 and the patient survey was approved on April 18, 2016. All respondents provided informed consent.

## Results

### Patients

A total of 699 patients were surveyed across six countries: Australia (*n* = 10), Canada (*n* = 64), Germany (*n* = 149), Italy (*n* = 106), Japan (*n* = 84), and the UK (*n* = 286). Of these patients, 174 were diagnosed with MF, 223 with PV, and 302 with ET. For MF and PV, the male to female ratio was similar (MF, 51% male; PV, 53% male), whereas, as expected, a greater proportion of patients with ET were female (68%) (Table 1). Men were generally older than women (mean age, 59.0 vs 55.5 years; *P* < .001), and patients with MF or PV were older than patients with ET (mean ages, 59.6, 57.9, and 54.9 years, respectively; *P* = .035). Physicians (*n* = 219) were from the same countries; most were hematologists (54%) or hematologists-oncologists (27%).

**Table 1:** Baseline characteristics

	MF (*n* = 174)	PV (*n* = 223)	ET (*n* = 302)	Total (*N* = 699)	*P* value*
Country breakdown, *n* (%)					< .01
Australia	5 (3)	4 (2)	1 (0.3)	10 (1)	
Canada	28 (16)	18 (8)	18 (6)	64 (9)	
Germany	57 (33)	50 (22)	42 (14)	149 (21)	
Italy	31 (18)	35 (16)	40 (13)	106 (15)	
Japan	8 (5)	38 (17)	38 (13)	84 (12)	
United Kingdom	45 (26)	78 (35)	163 (54)	286 (41)	
Patient age, mean (range), years	59.6 (28–89)	57.9 (20–85)	54.9 (18–86)	57.0 (18–89)	.035
Sex, *n* (%)					< .01
Male	89 (51)	118 (53)	98 (32)	305 (44)	
Female	85 (49)	105 (47)	204 (68)	394 (56)	
Disease duration since diagnosis, mean (range), years	4.0 (0–81)	6.6 (0–67)	6.3 (0–33)	−	
Length of time experiencing symptoms before diagnosis, *n* (%)					.306
< 6 months	56 (32)	56 (25)	96 (32)	208 (30)	
6–12 months	48 (28)	51 (23)	71 (24)	170 (24)	
1–2 years	32 (18)	47 (21)	54 (18)	133 (19)	
> 2 years	38 (22)	69 (31)	81 (27)	188 (27)	
Patient-reported prognostic risk score, *n* (%)					
High risk	23 (13)	20 (9)	60 (20)	103 (15)	
Intermediate risk	50 (29)	27 (12)	48 (16)	125 (18)	
Low risk	26 (15)	57 (26)	67 (22)	150 (21)	
Not known	75 (43)	119 (53)	127 (42)	321 (46)	
Employment status, *n* (%)					< .01
Employed full time	24 (14)	63 (28)	86 (28)	173 (25)	
Employed part time	20 (11)	23 (10)	59 (20)	102 (15)	
Unemployed, seeking employment	1 (1)	3 (1)	4 (1)	8 (1)	
Unemployed, not seeking employment	6 (3)	2 (1)	5 (2)	13 (2)	
Retired	75 (43)	75 (34)	84 (28)	234 (33)	
Self-employed	14 (8)	26 (12)	27 (9)	67 (10)	
Homemaker	8 (5)	16 (7)	17 (6)	41 (6)	
Student	2 (1)	2 (1)	3 (1)	7 (1)	
Disability	13 (7)	8 (4)	9 (3)	30 (4)	
Sick leave	5 (3)	3 (1)	6 (2)	14 (2)	
Other	6 (3)	2 (1)	2 (1)	10 (1)	

*ET* essential thrombocythemia, *MF* myelofibrosis, *PV* polycythemia vera

* *P* value was calculated using a *χ* ^2^ test

Slightly more patients were recruited by patient organizations (57%) than by physicians (43%); patients < 58 years old (age quartiles Q1 and Q2) were more likely to be recruited by patient organizations. A greater proportion of women were recruited by patient organizations (64%) than by physicians (36%), whereas a slightly higher proportion of men were recruited by physicians (53 vs 47%).

Median disease durations for respondents with MF, PV, and ET were 4.0, 6.6, and 6.3 years, respectively; more patients with MF had been diagnosed within 2 years of experiencing symptoms (MF, 78%; PV, 69%; ET, 73%) (Table 1). Nearly one half of patients were not aware of their disease-specific prognostic scores (MF, 43%; PV, 53%; ET, 42%), and 12% of patients with PV reported an intermediate score, which is not recognized by international guidelines. Overall, 49% of patients were employed full or part time; 33% were retired.

### MPN symptoms

**Fig. 1:**
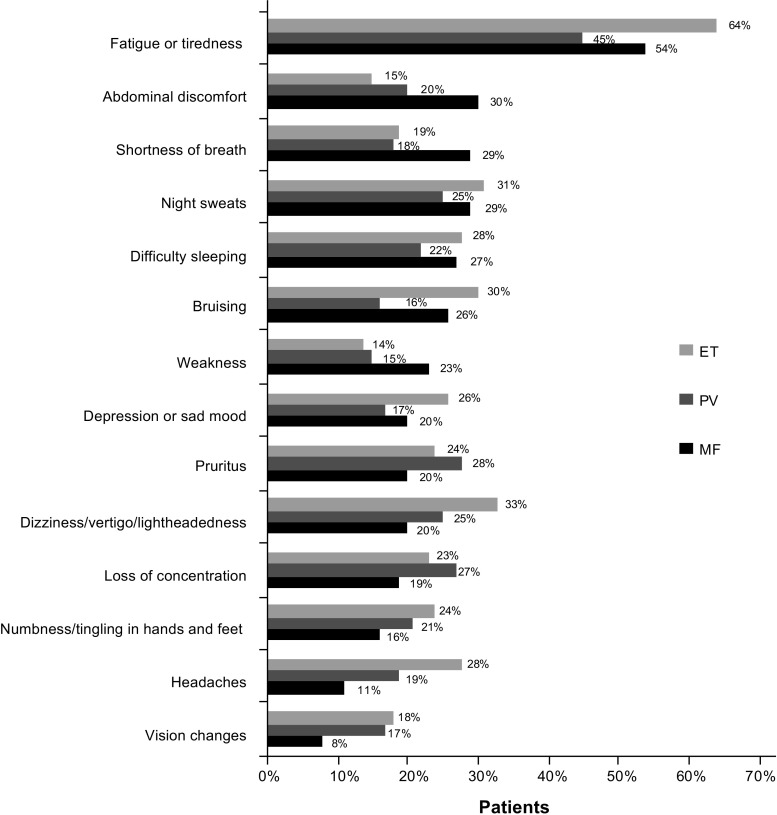
Symptoms experienced by patients in past 12 months. Top 10 symptoms for each disease are reported. *ET* essential thrombocythemia, *MF* myelofibrosis, *PV* polycythemia vera

Most patients (90%) experienced MPN-related symptoms in the past 12 months. In general, women reported having a higher overall symptom burden than men (lower quartile [Q1]: women, 45%; men, 55%; upper quartile [Q4]: women, 72%; men, 28%); no correlation was observed between age and overall symptom burden. Patients who were recruited for the survey via patient organizations reported a higher symptom burden than those recruited by physicians. Of those patients in the upper quartile (Q4), 91% were recruited by a patient organization and 9% by a physician. The most commonly reported symptom among all subtypes was fatigue (MF, 54%; PV, 45%, ET, 64%; Fig. 1), which was experienced by more women than men (61 vs 47%). Although fatigue was more prevalent in high-risk patients (74%), a substantial proportion of lower-risk patients also reported experiencing fatigue (intermediate risk, 47%; low risk, 47%). Similarly, the proportion of patients with fatigue was highest among patients in the most severe symptom burden groups (Q3, 74%; Q4, 95%); however, fatigue was still prevalent among patients experiencing a low symptom burden (Q1, 19%; Q2, 59%). The incidence of other common symptoms experienced in the past 12 months varied depending on disease subtype (MF: abdominal discomfort [30%], shortness of breath [29%], night sweats [29%], difficulty sleeping [27%]; PV: pruritus [28%], loss of concentration [27%], night sweats [25%], dizziness [25%]; ET: dizziness [33%], night sweats [31%], bruising [30%], difficulty sleeping [28%]). In general, low-risk patients reported experiencing fewer symptoms than high-risk patients. Overall, patients experienced an average of 5.8 symptoms at diagnosis, but this progressed to a significantly higher average of 6.9 symptoms after a median of 5.5 years after diagnosis (*P* < .001).

**Fig. 2:**
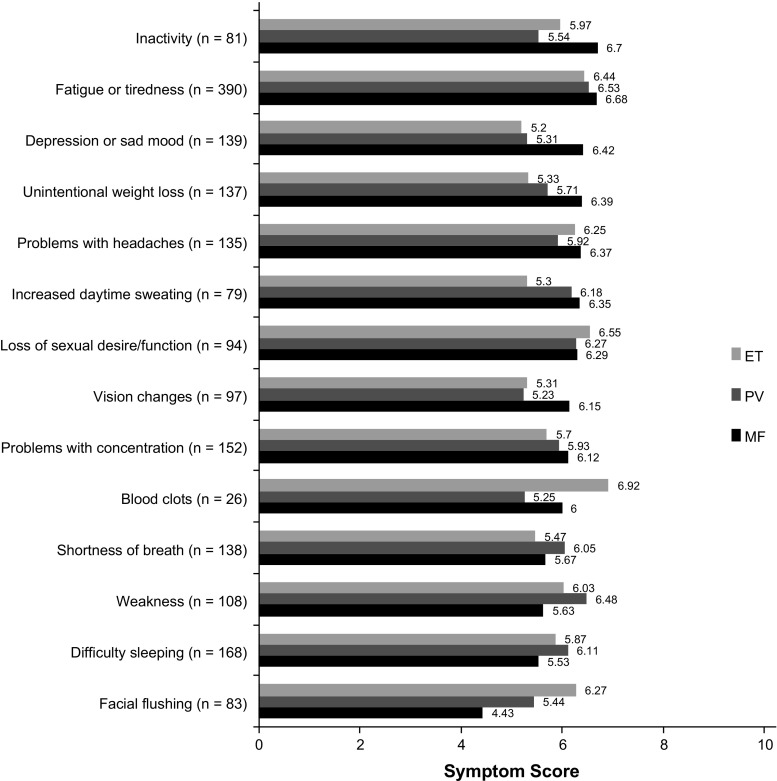
Symptoms reported as > 6 (for any disease) on a severity scale of 0 (not severe at all) to 10 (worst imaginable). Scores for which *n* < 20 are not presented. *ET* essential thrombocythemia, *MF* myelofibrosis *PV* polycythemia vera

Symptom severity was measured using a 0–10 severity scale based on the MPN symptom assessment form total symptom score (MPN-SAF TSS) [15]. Fatigue featured prominently on the list of most severe symptoms as reported by patients (mean severity score for patients experiencing the symptom: MF, 6.68; PV, 6.53; ET, 6.44), along with inactivity (mean severity score: MF, 6.70; PV, 5.54; ET, 5.97) (Fig. 2). Patients with ET reported a mean severity score of 6.92 for blood clots. The severity score for the overall MPN population was > 5 for 24 of 31 individual symptoms assessed. Scores > 5 for individual symptoms in the MPN-SAF TSS have been associated with advanced disease and the need for therapy [28, 29].

When asked which symptom they would most like to have resolved, most patients referred to improvement in fatigue/tiredness across all disease subtypes (MF, 86%; PV, 84%; ET, 77%), as well as blood clot dissolution (MF, 50%; PV, 75%; ET, 75%). Other symptoms most patients wanted to resolve included bone pain in patients with MF (58%), cerebral strokes and pruritus in patients with PV (67 and 63%, respectively), and cerebral strokes and headaches in patients with ET (67 and 58%, respectively).

### QOL and activities of daily living

**Fig. 3:**
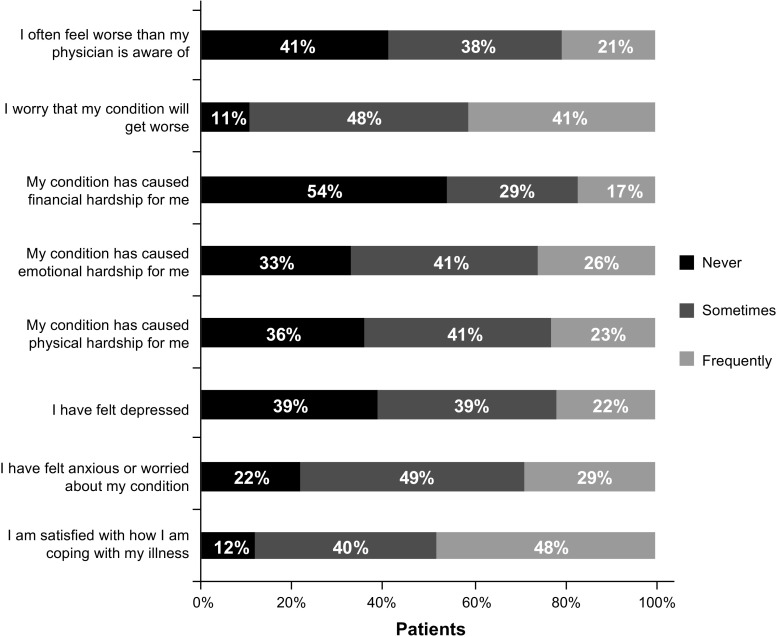
Patient impact ratings against select statements about disease impact on QOL. Patients were asked to “Rate the following statements as they have occurred during the past month, as a result of your condition.” Statements were ranked from 1 (not at all) to 5 (a great deal). For the purpose of this analysis, 1 = never, 2–3 = sometimes, and 4–5 = frequently. *QOL* quality of life

The majority of patients indicated that they experienced a reduction in QOL due to MPN symptoms (MF, 83%; PV, 72%; ET, 74%; Table 2). The proportion of patients indicating that they experienced a reduced QOL was highest among those with higher-risk scores (MF, 78%; PV, 70%; ET, 85%) and those with high symptom burden (Q4; MF, 93%; PV, 94%; ET, 91%). However, a substantial proportion of patients with low symptom burdens (Q1) reported reduced QOL (MF, 70%; PV, 56%; ET, 57%) as did those with low-risk scores (MF, 73%; PV, 53%; ET, 46%). Emotional burden associated with MPNs was rated on a scale of 1–5, for which 1 was “not at all” and 5 was “a great deal.” Overall, 26% of all patients stated that the disease frequently caused emotional hardship (mean score, 2.45), with 29% frequently feeling anxiety/being worried (mean score, 2.69) (Fig. 3). Additionally, 89% of patients worried that their condition would worsen. In general, women reported a higher burden than men. For example, female patients with MF felt a higher impact of the anxiety and worry they experienced over their condition (3.09 vs 2.69), emotional hardship (2.88 vs 2.44), and worry that their condition would worsen (3.34 vs 3.06).

**Table 2:** MPN symptom impact on QOL*

Symptoms reduce my life quality, %	MF (*n* = 151)	PV (*n* = 181)	ET (*n* = 253)	Total (*n* = 585)
Agree strongly	36	27	26	29
Somewhat agree	47	45	48	47
Somewhat disagree	11	14	14	13
Strongly disagree	6	14	11	11

*ET* essential thrombocythemia, *MF* myelofibrosis, *PV* polycythemia vera, *QOL* quality of life

*Includes patients experiencing symptoms

In general, responses were similar across all three MPNs. When assessed by MPN, 33, 14, and 23% of patients with MF, PV, and ET, respectively, expressed that their condition had caused emotional hardship; 34, 29, and 26% of patients reported that they had felt worried or anxious about the disease. The only significant differences were observed between patients with MF or ET, with patients with MF reporting higher mean scores for feeling anxious or worried (*P* = .02), physical hardship (*P* < .001), and emotional hardship (*P* = .005). Overall, 61% of patients felt some level of depression during the last month due to their condition, with 22% indicating that depression had a high impact on them. Approximately 10% of patients had received antidepressants to help manage their condition (MF, 11%; PV, 11%; ET, 7%). A similar proportion had received psychological therapy (MF, 9%; PV, 6%; ET, 8%). Interestingly, almost one-half of all patients (48%) indicated that they were frequently satisfied (score of 4–5 of 5) with how they were coping with the illness.

**Fig. 4:**
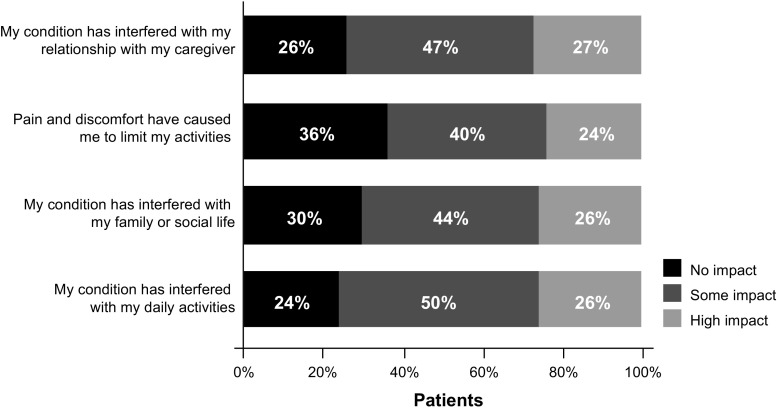
Patient impact ratings of select statements about disease impact on daily activities. Patients were asked “To what extent does your condition interfere with the following activities in your life?” Statements were ranked from 1 (not at all) to 5 (a great deal). For the purpose of this analysis, 1 = no impact, 2–3 = some impact, and 4–5 = high impact

In addition to causing emotional hardship, MPNs were also reported to have a high impact on daily activities, with approximately one quarter of patients reporting interference with daily activities (26%) or pain and discomfort that limited daily activities (24%) (Fig. 4). Furthermore, patients also reported that MPNs had a high impact on their relationship with their caregiver (27%) and interfered with family or social life (26%). Women also reported a higher burden in these areas compared with men, with the exception of their relationship with their caregiver.

A substantial proportion of patients (40%) reported requiring a caregiver (Table 3). When assessed by disease subtype, approximately one third of patients with PV (34%) or ET (33%) required assistance from a caregiver; however, this was significantly higher in patients with MF (58%; *P* < .001). Patients classified with high- or intermediate-risk disease were more likely to rely on someone for caregiving (53 and 47%, respectively) than those classified with low-risk disease (25%). Similarly, patients with a greater symptom burden were more likely to require a caregiver (Q1, 30%; Q4, 67%). Of those who reported requiring a caregiver, 68% stated that a spouse was their main caregiver, 17% stated that it was their son or daughter, and only 1% stated that it was a paid professional. Common tasks for which patients required the help of a caregiver included homemaking (61%), companionship (56%), and transportation (50%). On average, patients who required a caregiver received help for 12.3 h in the 7 days preceding the survey.

**Table 3:** Caregiver requirements*

*n* (%)	MF (*n* = 174)	PV (*n* = 223)	ET (*n* = 302)	Total (*N* = 699)
Never	73 (42)	148 (66)	201 (67)	422 (60)
Rarely	46 (26)	33 (15)	43 (14)	122 (17)
Sometimes	34 (20)	27 (12)	45 (15)	106 (15)
Often	21 (12)	15 (7)	13 (4)	49 (7)

*ET* essential thrombocythemia, *MF* myelofibrosis, *PV* polycythemia vera

*Patients were asked, “How often do you rely on someone to assist you with your activities of daily living due to your condition?”

### MPN impact on employment

Patients also reported a high impact on the ability to work. Of all patients, 9% voluntarily left their job, 8% took early retirement, 7% started receiving disability living allowance, 5% moved to a lower-paying job, and 2% experienced involuntary loss of work (Table 4); 49% of all patients were employed at the time of this survey (Table 1). A negative impact on the ability to work was observed across symptom burden quartiles; however, those with the highest overall symptom burden (Q4) experienced a greater negative impact than those with the lowest symptom burden (Q1): 18 vs 5% voluntarily left their job; 21 vs 4% took early retirement; 21 vs 4% received disability living allowance; 10 vs 3% moved to a lower-paying job, and 7 vs 0% experienced involuntary loss of work. A similar trend was observed across prognostic risk scores, with high-risk patients experiencing a greater negative impact on the ability to work than low-risk patients. On average over the past 7 days, employed patients with MF had missed 4.8 h of work, patients with PV 3.3 h, and patients with ET 2.6 h. Of the patients who were employed full time or part time at the time of the survey (MF, *n* = 44; PV, *n* = 86; ET, *n* = 145), ≈ 35% had missed hours of work within the past 7 days; this was highest in patients with MF (MF, 45%; PV, 31%; ET, 33%) (Table 5).

**Table 4:** Impact of MPN on work*

*n* (%)	MF (*n* = 174)	PV (*n* = 223)	ET (*n* = 302)	Total (*N* = 699)
Reduced hours at work	36 (21)	33 (15)	70 (23)	139 (20)
Voluntarily terminated your job	14 (8)	14 (6)	35 (12)	63 (9)
Been involuntarily terminated from job	3 (2)	7 (3)	4 (1)	14 (2)
Gone on disability living allowance	21 (12)	9 (4)	21 (7)	51 (7)
Taken early retirement	19 (11)	12 (5)	27 (9)	58 (8)
Taken a lower paid job	5 (3)	8 (4)	20 (7)	33 (5)

*ET* essential thrombocythemia, *MF* myelofibrosis, *MPN* myeloproliferative neoplasms, *PV* polycythemia vera

*Patients were asked, “As a result of your condition, have you ever . . .” Percentages represent those who responded “Yes”

**Table 5:** Work and activity impairment

All patients	MF (*n* = 174)	PV (*n* = 223)	ET (*n* = 302)	Total (*N* = 699)
Overall activity impairment	44.9	40.3	36.3	39.7
Employed patients	MF (*n* = 44)	PV (*n* = 86)	ET (*n* = 145)	Total (*n* = 275)
Absenteeism	11.7	5.9	7.4	7.6
Presenteeism (i.e., working while sick)	35.2	29.6*	30.7^†^	31.1
Overall work impairment	41.4	33.0*	35.7^†^	35.8
Hours missed from work, *n* (%)				
Mean, hours^‡^	4.8	3.3	2.6	3.1
SD	3.71	17.25	6.52	10.27
1–3 h	4 (9)	8 (9)	18 (12)	30 (11)
4–6 h	8 (18)	9 (10)	9 (6)	26 (9)
7–9 h	3 (7)	4 (5)	14 (10)	21 (8)
> 10 h	5 (11)	6 (7)	7 (5)	18 (7)

*ET* essential thrombocythemia, *MF* myelofibrosis, *PV* polycythemia vera

*n = 83

^†^ *n* = 140

^‡^Mean scores calculated using Work Productivity and Activity Impairment scoring

(http://www.reillyassociates.net/WPAI_Scoring.html)

Across all MPN subgroups, a substantial proportion of patients reported overall impairment at work (mean among currently employed patients: MF, 41.4%; PV, 33.0%; ET, 35.7%) and in overall activity (mean among all patients: MF, 44.9%; PV, 40.3%, ET, 36.3%) (Table 5). As before, those with a higher symptom burden experienced a greater negative impact on work productivity. Overall work impairment was reported in 56.0 vs 30.6% of patients with the highest and lowest symptom burdens, respectively; 59.1 vs 32.1% of patients reported impairment in overall activity. Patients with MF experiencing the highest symptom burden had the greatest overall work impairment (mean: MF, 62.8%; PV, 48.3%; ET, 58.4%) and overall activity impairment (mean: MF, 65.5%; PV, 57.3%; ET, 56.7%). Similarly, patients with higher-risk scores also experienced a greater negative impact on work productivity; 42.5% high-risk vs 30.2% low-risk patients reported overall work impairment and 45.6 vs 31.9% reported overall activity impairment. Overall, 54% of caregivers were employed, and 13% of those had to reduce their hours at work to care for an individual. Additionally, 6% considered terminating their job or moving to part-time work, 5% took early retirement, 4% voluntarily terminated their job, and 3% were involuntarily terminated from their jobs.

### Disease management

Most patients were receiving therapy for MPN. Overall, 72, 68, and 72% of physicians were likely to recommend drug treatment for patients with MF, PV, and ET, respectively, who were experiencing severe symptoms. Similarly, 71, 61, and 39% of physicians were likely to recommend treatment for patients with MF, PV, and ET, respectively, who were experiencing symptomatic splenomegaly. Overall, 43% of physicians assessed symptoms by proactively asking patients how they were feeling; 37% asked about specific symptoms and 11% waited for patients to mention any bothersome symptoms. However, 69% of physicians reported that they always assessed symptom presence or severity at every visit. When discussing symptoms, 49% of physicians discussed those most likely experienced by their patients, 32% discussed the most bothersome symptoms, and 17% went through a comprehensive list; 2% of physicians did not discuss symptoms with their patients. Interestingly, only 26% of physicians used a validated symptom assessment form; 44% used their own rating method.

Main therapies ever received by patients included ruxolitinib (54%), aspirin (40%), and hydroxyurea (HU; 28%) in MF; phlebotomy (70%), aspirin (66%), and HU (42%) in PV; and aspirin (73%), HU (48%), and anagrelide (15%) in ET. Physicians reported currently prescribing ruxolitinib (76%), transfusion (54%), and HU (53%) to manage patients with MF; aspirin (79%), HU (77%), and phlebotomy (67%) for PV; and aspirin (80%), HU (67%), and anagrelide (52%) for ET. All other therapies were prescribed by < 50% of physicians.

The majority of patients with PV (70%) reported having been treated with phlebotomy. Of those who had received phlebotomy (*n* = 155), 71% were very or somewhat satisfied and 25% were very or somewhat dissatisfied; 25% thought that phlebotomies had a high negative impact on their QOL. Similarly, 37% of physicians thought that phlebotomies had a high negative impact on patient QOL; 56% thought that phlebotomies had some degree of burden. Additionally, physicians reported that phlebotomy alone was insufficient to control the condition in 38% of their patients. Overall, patients stopped phlebotomies because their physician decided it was no longer necessary (62%), patients felt worse after treatment (10%), and the frequency of visits was inconvenient (8%). Physician-reported reasons for stopping phlebotomies were that frequency of visits was inconvenient (38%), patients felt worse after treatment (35%), and lack of intravenous access (33%).

In addition to phlebotomy, the use of HU was also assessed in patients with PV or ET; use of HU in patients with MF was not assessed. Of those who received HU (PV, *n* = 95; ET, *n* = 145), 78 and 74%, respectively, continued to receive HU; 19 and 22% were somewhat or very dissatisfied with HU therapy. Main reasons for stopping HU were lack of efficacy (PV, 29%; ET, 13%) and toxicity (PV, 19%; ET, 27%). Overall, 78% of physicians reported that up to 25% of their patients showed inadequate efficacy or intolerance of HU.

Interestingly, many physicians (MF, 51%; PV, 47%; ET, 49%) chose watchful waiting to manage > 25% of their patients at diagnosis. Overall, patients who were still being managed with watchful waiting at the time of this survey (*n* = 44) had a low (Q1-Q2) overall symptom burden; however, 23% had a moderate to high (Q3-Q4) overall symptom burden.

**Fig. 5:**
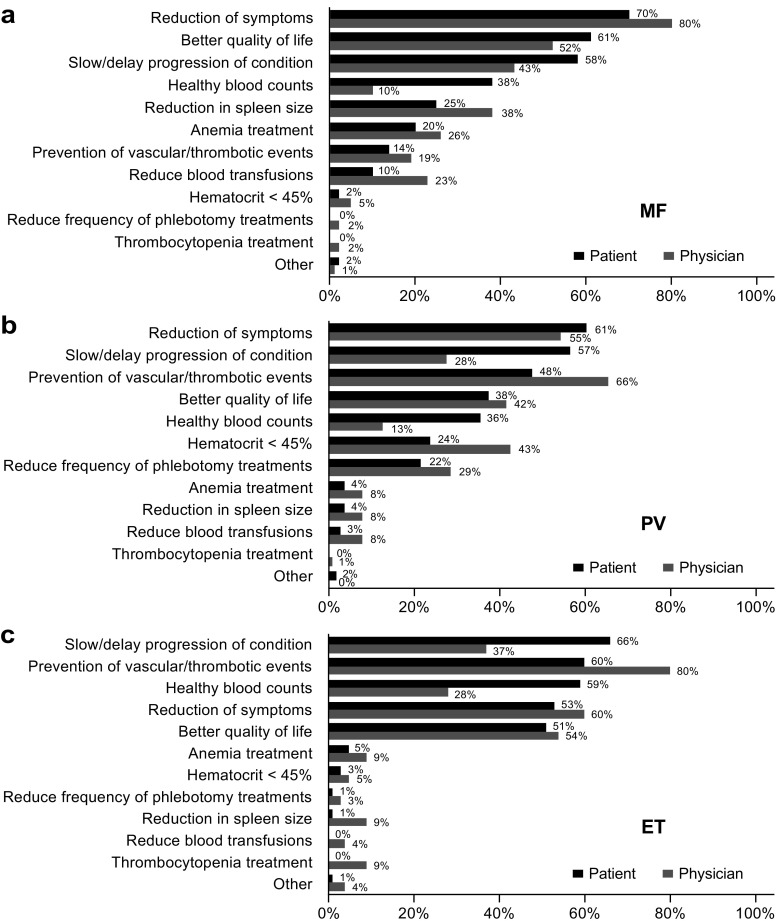
Most important treatment goals in **a** MF, **b** PV, and **c** ET as reported by patients and physicians. Patients were asked, “Other than a cure for your condition, what are your 3 most important treatment goals? Please assign rankings (1-3), with 1 being the most important.” Physicians were asked, “Other than a cure, what is your most important treatment goal for therapy for each disease? Starting with 1 as the most important, 2 as the second, and 3 as the third, please write 1, 2, and 3 for each disease.” The figure shows the proportion of patients and physicians who selected the “goal” within their top 3

Consistent with the impact of symptom burden on patient lives, patients and physicians were both concerned about reducing symptoms (patients: MF, 70%; PV, 61%; ET, 53%; physicians: MF, 80%; PV, 55%; ET, 60%); however, patients were also concerned about delaying MPN progression (MF, 58%; PV, 57%; ET, 66%; physicians: MF, 43%, PV, 28%; ET, 37%) (Fig. 5). Compared with patients, physicians indicated a greater focus on prevention of vascular/thrombotic events in PV (66 vs 48%) and ET (80 vs 60%). Overall, only 27% of physicians completely agreed with their patients on treatment goals; 66% “somewhat” agreed. However, most patients (87%) were satisfied with their physician’s disease management/communication.

Main measures of treatment success among patients were physician feedback (MF, 73%; PV, 75%; ET, 75%) and blood counts (MF, 72%; PV, 67%; ET, 74%). Symptom relief (MF, 41%; PV, 27%; ET, 26%) and improved QOL (MF, 40%; PV, 24%; ET, 27%) were also considered measures of treatment success, but to a lesser extent. Lack of efficacy (MF, 85%; PV, 80%; ET, 79%), side effects (MF, 60%; PV, 66%; ET, 61%), and disease progression (MF, 64%; PV, 58%; ET, 58%) were key reasons for changing therapies. Among physicians treating PV, 45% mentioned that inconsistent hematocrit control was also a key reason for change. Other reasons reported included change in symptoms, cytopenias, and patient preference.

## Discussion

The international MPN Landmark survey evaluated the impact of MPNs in a contemporary global cohort of patients. Findings from this large survey indicated that patients with MPNs experience a high disease burden. Patients had a high prevalence of symptoms, with fatigue being one of the most common and most severe symptoms. Symptoms were present despite the fact that most patients had received or were receiving treatment during the time of the survey. Additionally, patients experienced a reduction in emotional well-being, QOL, activities of daily living, and ability to work. These results are consistent with previous reports of symptom burden and QOL that included non-US patients [15, 25, 27], as well as the recent US Landmark survey [23].

The impact of MPN on employment and daily activities had not been assessed previously in patients outside of the USA. Findings from our study showed that MPNs greatly impacted patient work and productivity, with MF leading to higher rates of absenteeism. Patients not only went on medical disability, but many took early retirement or left the workforce; 20% of patients reduced their hours at work. This was consistent with what was observed in the US population. Although the reason is unclear, a lower proportion of non-US patients went on medical disability (7 vs 11%), took early retirement (8 vs 14%), left the workforce (11 vs 17%), or reduced work hours (20 vs 26%) compared with US patients. Interestingly, the proportion of patients who reported that the disease interfered with daily activities or with family or social life was higher in patients outside of the USA (daily activities, 76 vs 46%; family or social life, 70 vs 65%).

Although the majority of patients reported that the disease interfered with daily activities, only 40% of patients overall reported requiring a caregiver. Of note, a significantly higher proportion of patients with MF than with PV or ET relied on a caregiver. This was likely a reflection of the higher symptom burden observed in patients with MF in this and other studies [15], as well as other factors, such as weight loss or the need for transfusions.

Interestingly, our study showed that patients with low-risk scores may also experience high disease burden and reduced productivity. For instance, more than one half of patients with low-risk scores reported reduced QOL (MF, 73%; PV, 53%; ET, 46%), and approximately one third of low-risk patients had reduced work productivity (30.2% reported overall work impairment). Additionally, many of these patients also reported requiring caregivers. Currently, most low-risk patients are managed using a “watch-and-wait” approach [30, 31]; however, our findings indicated an unmet need in the management of low-risk patients with MPNs. A new treatment strategy that leads to better QOL in this patient group, such as targeted therapy or psycho-oncological therapy, may be needed; however, risks associated with any new approaches will also have to be considered. Similarly, a substantial proportion of patients with low symptom burden reported reduced QOL (MF, 70%; PV, 56%; ET, 57%) and productivity (30.6% reported overall work impairment), suggesting that new treatment strategies may also benefit these patients.

Findings from this study also showed that patients with ET have a high symptom burden, with most of these patients reporting an impact on QOL. For example, fatigue was present in > 60% of patients and several symptoms were considered severe. Additionally, symptom burden led to work productivity impairment in a substantial proportion of patients with ET, with 23% of patients reducing their hours at work. These findings are consistent with those of the US MPN Landmark survey and together suggest that symptom improvement may need to play a more central role in the management of ET.

In addition, our study suggests a need for proactive and standardized symptom assessment at diagnosis and over the course of treatment to ensure that patients receive optimal therapy. For instance, although most patients in our survey received treatments that were in line with current treatment guidelines, a large proportion (23%) of patients managed with watchful waiting had a moderate to high symptom burden yet did not receive any drug therapy. However, cytoreductive treatment is recommended for patients experiencing disease-associated symptoms, regardless of risk group [31]. This may be a result of physicians using different ways of assessing symptom severity, suggesting a need for a standardized assessment of symptoms during patient visits.

Alternatively, this could also be due to a proportion of physicians not recognizing symptoms as a reason for treating patients with MPNs. For example, only 72% of physicians would treat patients with severely symptomatic MF despite current guidelines recommending cytoreductive therapy for these patients. Additionally, our study showed that patients with high symptom burden, including those with low-risk disease, have reduced QOL (MF, 93%; PV, 94%; ET, 91%) and productivity (mean overall work impairment: MF, 62.8%; PV, 48.3%; ET, 58.4%) and would likely benefit from treatment. However, we acknowledge that the choice to treat should be determined on an individual basis and would ultimately depend on the risk-benefit balance of therapy in each patient.

Limitations of our analysis included the descriptive nature of the study and self-reporting of clinical information by patients. Because the study was designed to be analyzed descriptively, no statistical comparisons of the data were possible. Additionally, approximately one half of all patients did not know their prognostic scores, making it difficult to interpret responses by prognostic risk group. Furthermore, online administration of the survey may have biased the patient population to include only patients with a certain level of education and/or financial means that would allow them to understand and take an internet-based survey. Furthermore, physicians and patients were recruited independently and responses were not linked.

Recruitment procedures may have also biased the results. Recruitment of patients was carried out via either physicians or local patient organizations. In instances when patient organizations were used (UK, Canada, and Germany), patients may have been part of a more engaged population; for example, a higher number of symptoms were observed among patients recruited via a patient organization. This was especially observed in countries where a large patient organization population was engaged (UK and Canada) in comparison with those recruited via physicians.

Overall, our study showed that patients with MPNs have severe disease burden, reduced QOL, and impaired productivity, regardless of geographic location. Findings from our study suggest that managing disease burden in patients with MPNs is crucial to minimize disease impact on patient daily lives. Treatment for MPNs should therefore include therapies that can reduce symptom burden and improve QOL. These are important considerations as targeted or psycho-oncological therapies continue to be evaluated and developed. The study also revealed a lack of alignment between physician and patient perceptions relating to communication and disease management, as well as a lack of standardization in symptom assessment. Of note, patients often had different treatment goals than physicians, indicating a need for improved patient-physician communication and a treatment plan that includes proactive and standardized monitoring of symptoms and agreement on treatment goals. Further analyses on physician and patient interactions, country differences, and treatment patterns will be important in shaping and improving the management of these patients.
